# Therapeutic Efficacy of Artemether-Lumefantrine (Coartem®) in Treating Uncomplicated *P*. *falciparum* Malaria in Metehara, Eastern Ethiopia: Regulatory Clinical Study

**DOI:** 10.1371/journal.pone.0154618

**Published:** 2016-04-29

**Authors:** Desalegn Nega, Ashenafi Assefa, Hussein Mohamed, Hiwot Solomon, Adugna Woyessa, Yibeltal Assefa, Amha Kebede, Moges Kassa

**Affiliations:** 1 Malaria and Other Vector-Borne Parasitic Diseases Research Team, Bacterial, Parasitic and Zoonotic Diseases Research Directorate, Ethiopian Public Health Institute, Addis Ababa, Ethiopia; 2 Malaria research team, disease prevention and control directorate, Federal Ministry of Health, Addis Ababa, Ethiopia; Université Pierre et Marie Curie, FRANCE

## Abstract

**Background:**

As per the WHO recommendation, the development of resistance by *P*. *falciparum* to most artemisinin combination therapies (ACTs) triggered the need for routine monitoring of the efficacy of the drugs every two years in all malaria endemic countries. Hence, this study was carried out to assess the therapeutic efficacy of Artemether-Lumefantrine (Coartem®) in treating the uncomplicated falciparum malaria, after 9 years of its introduction in the Metehara, Eastern Ethiopia.

**Method:**

This is part of the therapeutic efficacy studies by the Federal Ministry of Health Ethiopia, which were conducted in regionally representative sentinel sites in the country from October 2014 to January 2015. Based on the study criteria set by WHO, febrile and malaria suspected outpatients in the health center were consecutively recruited to study. A standard six-dose regimen of AL was administered over three days and followed up for measuring therapeutic responses over 28 days. Data entry and analysis was done by using the WHO designed Excel spreadsheet and SPSS version 20 for Windows. Statistical significant was considered for *P-value* less than 0.05.

**Result:**

Of the 91 patients enrolled, the day-28 analysis showed 83 adequate clinical and parasitological responses (ACPRs). Per protocol analysis, PCR-uncorrected & corrected cure rates of Coartem® among the study participants were 97.6% (95%CI: 93.6–99.5) and 98.8% (CI: 93.5–100%), respectively. No parasite detected on day 3 and onwards. Fever clearance was above 91% on day-3. Mean hemoglobin was significantly increased (P<0.000) from 12.39 g/dl at day 0 to 13.45 g/dl on day 28. No serious adverse drug reactions were observed among the study participants.

**Conclusion:**

This study showed high efficacy of AL in the study area, which suggests the continuation of AL as first line drug for the treatment of uncomplicated *P*. *falciparum* malaria in the study area. This study recommends further studies on drug toxicity, particularly on repeated cough and oral ulceration.

## Introduction

*P*. *falciparum* resistance to chloroquine and sulphadoxine-pyrimethamine (SP) in most African countries, including Ethiopia, has triggered the shift of national treatment policy towards the first-line treatment with ACTs [1,2]. WHO currently recommends five ACTs: Artesunate-sulfadoxine-pyrimethamine, Artesunate-mefloquine, Artesunate-amodiaquine, Artemether-Lumefantrine (AL) and Dihydroartemisinin-Piperaquine (DHA-PPQ). The first 3 regimens are no more used in Africa because of safety concerns and the emergence of drug resistance and only AL has been the most widely prescribed ACT in sub-Saharan Africa [2,3]. In Ethiopia, using AL as the first line treatment for uncomplicated falciparum malaria has been started in 2004 [1,4].

*P*. *falciparum* resistance to oral artemisinin monotheraphy has been reported severally along the Cambodia–Thailand border in the Greater Mekong sub-region of Asia [5,6]. Despite the truth, combination therapies are still remained the first choice [3,7] and there is no relevant report of ACT resistant *P*. *falciparum* detected in Africa, including Ethiopia [8–10]. ACT is known by its rapid parasite clearance and reducing gametocyte carriage to cease parasite transmission [11–13].

AL (Coartem®) is a first fixed-dose ACT, which meets the WHO prequalification criteria for efficacy, safety and quality, and is indicated for the treatment of uncomplicated falciparum malaria, or mixed infections in adults, children, and infants (>5 kg body weight). AL confirmed the reliable 28-day polymerase chain reaction (PCR)-corrected cure rates of more than 95% in adult and pediatric populations with a favorable safety and tolerability in sub-Saharan African studies [14,15].

Malaria trends change over time; thus, routine monitoring of the efficacy of the therapeutics-in-use is essential for timely changes in treatment policy and helps to detect early changes in *P*. *falciparum* sensitivity to antimalarial drugs as recommended by WHO [16]. WHO currently recommends routine monitoring of the efficacy of first-line and second-line ACTs every two years in all endemic countries. A treatment failure rate exceeding 10% should prompt a change in the national antimalarial treatment policy [16]. Furthermore, there was no similar study conducted in the study area previously. Therefore, this study was conducted with the aim of assessing the therapeutic efficacy of Artemether-Lumefantrine (Coartem®) in treating the uncomplicated falciparum malaria, after 9 years of its introduction in Metehara, Eastern Ethiopia.

## Materials and Methods

### Study Area and Period

This study was conducted in between October 2014 and January 2015 during the peak malaria transmission season at the outpatient department (OPD) of Metehara Health Centre. Metehara is an administrative town of Fentale district in Oromia Regional state of Ethiopia. The area is located in the Great Rift Valley, about 210 km east of Addis Ababa, the country’s capital. Its coordinate is 8°54′N 39°55′E and average elevation is 947 m (3,107 ft), with time zone of EAT (UTC+3). Rivers Awash and Germama, and lake Basaka are important water bodies in the district [17]. The estate irrigation by using the nearby rivers for the industrial cultivation of sugar cane, in turn, suits the condition for breeding of the *Anopheles* mosquito. Transmission of malaria in this area occurs year-round, with peaks from September to November and March to May. The dominant parasites are *P*. *falciparum* causing 80% of malaria infections and *P*. *vivax* accounting for 20% infections (Metehara district health records, unpublished data).

### Study Design

This is part of the open-label single-arm 28-day regulatory clinical studies by the Federal Ministry of Health Ethiopia, which were conducted in regionally representative sentinel sites in the country, for the routine monitoring of the national treatment policy.

### Study Subjects, Inclusion, and Exclusion Criteria

Febrile patients visiting the outpatient department of the health center who fulfilled the inclusion criteria set by WHO [16], for the assessment and monitoring of antimalarial drug efficacy for the treatment of uncomplicated falciparum malaria.

#### Inclusion criteria

Both sex with age ≥6 months; body weight >5 kg; fever (axillary temperature ≥ 37.5˚c) or having history of fever in the preceding 24 hours; microscopically defined *P*. *falciparum* mono-infection, with parasitemia from 1000 to 100,000 parasites/μl; non-pregnant or not breast-feeding women; patients living within facility catchment area (i.e. 5–10 km radius of the health center); informed consent by patient or by caregivers for children and patient agreement to return for all scheduled visits.

#### Exclusion criteria

Mixed or mono-infection with species other than *P*. *falciparum*; hemoglobin >5.0 gm/dl; AL intake within 15 days prior to study recruitment; unable to take oral medication or repeated vomiting; known hypersensitivity to AL; evidence of severe malaria or other danger signs such as inability to drink or breast-feed, vomiting (i.e. more than twice in past 24 hours), recent history of convulsions (i.e. more than once in past 24 hours), unconscious state, unable to sit or stand; severe malnutrition; any history of previous serious side-effects to study medications; evidence of a concomitant febrile illness (Otitis media, tonsillitis, measles, abscesses, measles, acute lower respiratory tract infection, severe diarrhea) in addition to malaria

### Sample Size Determination

The required sample size was calculated by using a single population proportion formula based on the revised WHO protocol [16]. By taking an expected the day 28 cure rate of AL as 95% from estimation based on recent studies in the home country [8,18,19], a 95% confidence level and 5% margin of error, the initial calculated sample size was 73. Assuming an additional 20% contingency for withdrawals, a minimum of at least 88 representative sample size was computed.

### Sampling Technique and Sample Collection

Consecutive sampling technique had been used to select the visiting outpatients until the required sample size was attained. Patient screening was done by health officers in an outpatient setting to identify those meeting the inclusion criteria for the study. Health professionals involved in the collection of the data were oriented on the procedures designed by the WHO [16]. The finger-prick capillary blood was used to prepare the malaria slide smears, to measure hemoglobin level and to collect the dried blood samples on filter paper during enrolment and on any unexpected visit, for further molecular analysis.

### Blood Film Examination

Thick blood film was used to detect malaria parasites and to quantify the parasites, while thin film was used to identify the parasite species. Giemsa working solution with buffering PH of 7.2 was used to stain the smears. Double-slide blood smears were prepared; one stained rapidly with 10% Giemsa for 10 to 15 minutes to screen for recruitment, and the next stained with standard 3% Giemsa for 30 to 45 min as recommended elsewhere [16].

The first blood smear was used for parasite detection and rapid estimation of asexual parasitemia in limited microscopic fields. Parasitemia was taken as adequate for recruitment when ≥1 parasite/6–8 WBCs was observed; which matches to about 1000 asexual parasites/*μL*. The next standard blood smear was examined to determine the actual parasite density. Asexual parasite density per micro liter (μl) was determined on the basis of the number of parasites counted per 200 white blood cells on a thick blood film by assuming a total standard WBC count of 8000/μl On another hand, gametocytes were counted per 1000 WBC, based on the standard WBC count of 8000/μl [20,21].

Two experienced laboratory technologists individually examined the microscopic slides. Thick smears were reported as negative when no parasite detected after examining 100 microscopic fields. Discrepancy between the first and second readings was settled by a third senior microscopist in regional malaria examination quality control laboratory, and whose readings were then taken final. Quality control of microscopic results of parasite counts was made by crosschecking all of the total slides [20,21].

### Hemoglobin Measurement

Finger prick capillary blood was used to measure hemoglobin level using the Portable Spectrophotometer (Hemocue Hb 201+, Anglom, Sweden).

### Treatment and Follow Up Schedule

A standard six-dose regimen of AL(Coartem®) based on age/weight scale was administered twice daily over three days and was followed up for clinical and parasitological responses over 28 days based on the in-vivo drug trial protocol set by the WHO in 2009 [16]. AL was given as a tablet of 20 mg of artemether and 120 mg of lumefantrine. The AL was made in India by IPCA Laboratories Ltd. [Plot No: 255/1; Athal, Silvassa 396 230 (D & NH); Batch No: DYI473602; Mfg: 08–2013; and Exp: 07–2015]. The drug was provided by the FMOH Ethiopia through the support of WHO.

Patients were awaited for 30 min in the OPD to ensure absence of post-treatment vomiting. In vomiting cases, full-treatment dose was given again in the OPD. For repeated vomiting, patients were withdrawn from the study totally. Three doses were given on direct observation by the health officers in the OPD: first dose at recruitment and the next two morning doses given during visits at days 1 & 2. The rest three doses were taken at the patients’ home with the supervision of community health extension workers. Artisunate injection was given to patients who failed to respond to AL and those with re-appearance of *P*. *falciparum*. *P*. *vivax* case detected during follow-up was given the usual chloroquine.

Follow up visits for measuring therapeutic responses were made on days 1, 2, 3, 7, 14, 21, and 28 and/or any unscheduled day when symptoms occurred. The measurement of parasitemia, fever, and adverse events were made at every visit day; while hemoglobin was measured on days 0, 14, and 28. When patients failed to attend their scheduled visits, they were traced by the assigned home visitor on the same day and were brought to the clinic.

### Patient Withdrawal and Loss to Follow Up

Withdrawal of patients from the study was done in the conditions including self-willing, vomiting the drug twice, occurrence of a severe febrile illness, self-administration of drug with antimalarial effect, omitting the prescribed treatment doses, loss to follow up, detection of mixed *Plasmodium* species and other similar protocol violation situations. Patients who missed to attend on the follow-up schedules and/or could not attend even by tracing of the home visitor on the successive days were taken as *lost to follow-up*. Patients withdrawn by presence of complications including re-appearance of *P*. *falciparum or P*.*vivax* were admitted to the health center for proper inpatient treatment. Classification of therapeutic response and definitions

According to the WHO *in-vivo* drug trial protocol of 2009 [16], therapeutic responses were classified as primary and secondary outcomes. The primary endpoints were treatment failures and cure rates on day 28, while secondary endpoints were fever and parasitaemia clearance on days 1, 2 and 3; gametocytaemia carriage at days 7 and 28; and re-infection rates at day 28.

### The Primary Outcomes

#### Early treatment failure (ETF)

It is the development of danger signs for severe malaria on days 1, 2 or 3 in the presence of parasitemia; parasitemia on day 2 higher than day 0 count irrespective of axillary temperature; parasitemia on day 3 with axillary temperature ≥37.5 °C; parasitemia on day 3 ≥ 25% of count on day 0.

#### Late clinical failure (LCF)

It is the development of danger signs for severe malaria after day 3 in the presence of parasitemia, without previously meeting any of the criteria of ETF; presence of parasitemia and axillary temperature ≥37.5°C or history of fever on any day from day 4 to day 28, without previously meeting any of the criteria of ETF.

#### Late Parasitological Failure (LPF)

It is the presence of parasitemia on any day from day 7 to day 28 and axillary temperature *<*37.5°C, without previously meeting any of the criteria of early treatment failure or late clinical failure.

#### Adequate Clinical and Parasitological Response (ACPR)

It is the absence of parasitemia on day 28 irrespective of axillary temperature without previously meeting any of the criteria of ETF, LTF or LPF.

### Secondary Outcomes

#### Fever clearance rate (FCR)

It is the speed at which body temperature is decreased to *<*37.5°C after drug intake or proportion of patients whose fever cleared on days 1, 2, and 3.

#### Parasite clearance rate (PCR)

It is the speed at which parasites disappear from the body after drug intake or proportion of patients with negative blood smears on days 1, 2, and 3.

#### Gametocyte carriage

Proportion of patients with gametocytes during the course of the study.

#### Parasite Resistance

It was defined as the ability of a parasite strain to survive and/or multiply despite the administration and absorption of a drug in doses equal to or higher than those usually recommended but within the limits of tolerance of the subject.

#### PCR for identification of recrudescence

Genotyping of treatment failure samples was done to differentiate recrudescence from re-infection. PCR was conducted on standard filter paper collected blood samples, which were air-dried and stored in cool and dark boxes with desiccants. The analysis was performed at Ethiopian Public Health Institute (EPHI) malaria molecular laboratory similarly by using *msp-1 & msp-2* genes as described elsewhere [22,23]. Cases with identical genotypes for pre-and post-treatment samples were classified as recrudescence; and those with different genotypes as re-infection, while mixed genotypes were considered as failures.

### Ethical Consideration

#### Study approval

The study protocol was cleared and approved by the Ethical Review Committee of EPHI, Ethiopia.

#### Acquisition of consent

Verbal and written informed consents were obtained from all of the study participants; signed by the adults or the parent/guardian understanding in his/her local language. Data collected from each study participant and results of laboratory tests were kept confidential and used only for the research purpose.

### Data Analysis

Data entry and analysis was done by using the WHO designed Excel spreadsheet and SPSS version 20 for Windows. Descriptive statistics such as percentages, mean, standard deviation, and range were performed as needed. Cure rate was assessed by modified intention to treat (ITT) and per protocol (PP) analysis. The PP Kaplan Meir was used to analyze the primary therapeutic outcomes. Independent t-test was used to check the presence or absence of statistically significant mean differences between the continuous variables among age groups. Statistical significant was considered for *P-value* less than 0.05.

## Result of the Study

### Screening, Admission and Follow-Up of Patients

**Fig 1:**
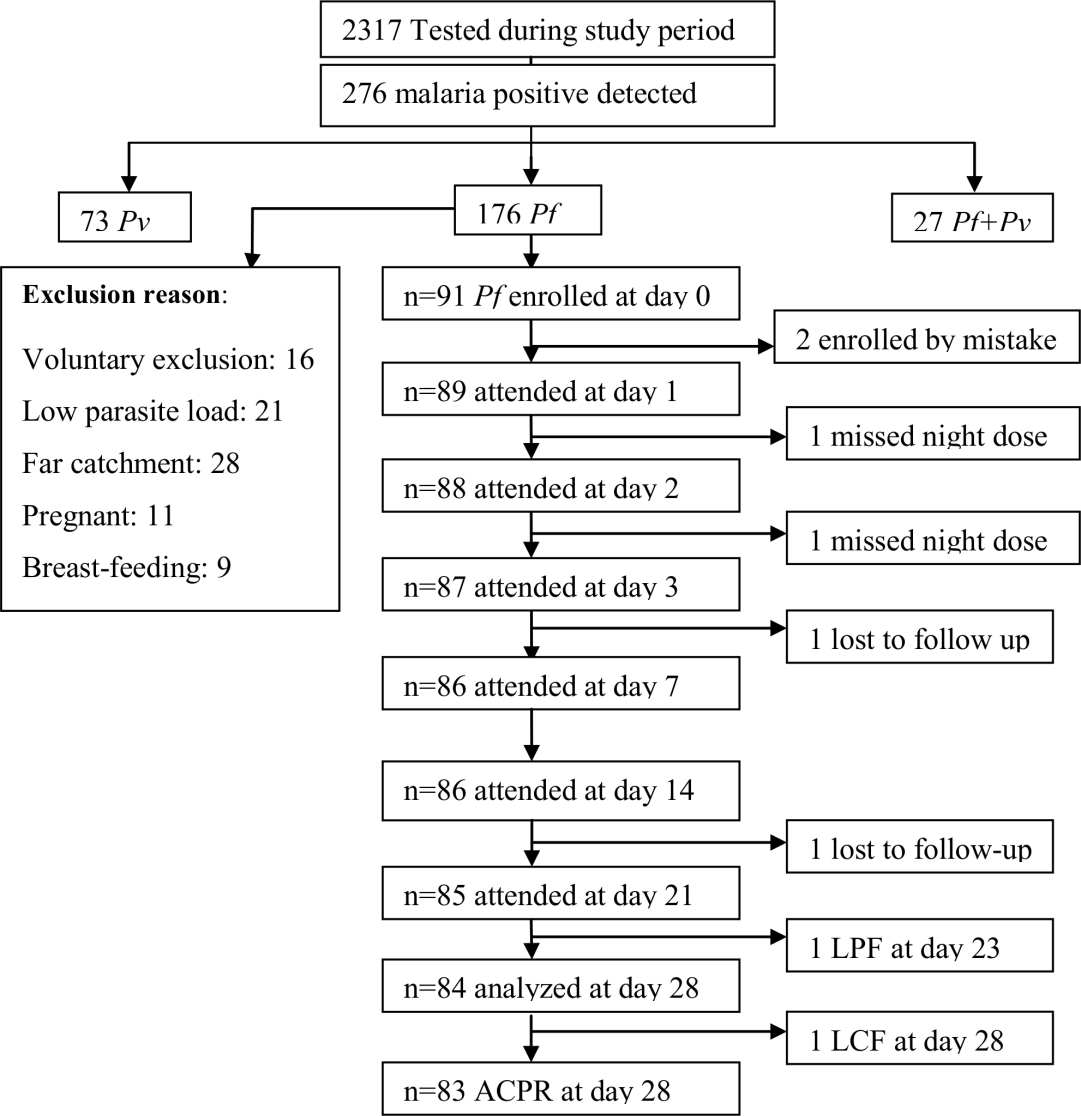
Flow chart showing screening of patients for Coartem® efficacy study, Metehara, Oct 2014-Jan 2015.

Outpatients who were febrile and having fever history within the 24 hrs prior to the start of the study were screened in outpatient department and sent to laboratory with the request of malaria diagnosis. Within the study period, 2317 patients clinically suspected for malaria were examined for the detection of *Plasmodium* parasite. Of the malaria suspected cases, 276 (11.9%) had malaria infection. From the malaria positives, 176 (63.8%) were *P*. *falciparum* mono-infections; of which 73(26.4%) were *P*. *vivax* infections and the rest 27(9.8%) were mixed infections. Of the *P*. *falciparum* mono-infection patients, only 91 patients who fulfilled the inclusion criteria for the surveillance of antimalarial drug efficacy set by WHO (16) were recruited to the study. The rest *P*. *falciparum* mono-infections cases were excluded for the reasons: voluntary exclusion, parasite load <1000 parasites/μl, residence far apart from the health centre, and pregnancy cases. More description is on the flow chart (Fig 1).

### Baseline Characteristics of the Study Participants

Totally 91 individuals were enrolled, of which 75.8% (69/91) were males; the proportion increased consistently with age. Mean age of the study participants was 18.4 years (range: 1–49); and mean age among the under five (U5), 5–15 & above 15 years was 2.6 yrs (1–4), 8.0 yrs (5.0–14.0) and 25.7 yrs (16–49), respectively. Majority of the study participants were adults (61.5%) as shown in the Table 1.

**Table 1:** Baseline characteristics of the study participants, Metehara, Oct 2014-Jan 2015.

	Age stratification			
Variables at enroll.	<5 years(n = 9)	5–15 years (n = 26)	>15 years (n = 56)	Total (n = 91)
Male sex	5(55.6)	19(73.1)	45(80.4)	69(75.8)
Mean age(yrs)	2.6(1–4)	8.0(5.0–14.0)	25.7(16–49)	18.40(1–49)
Weight(kg)	16.2(9–61)	23.4(13.5–41.0)	53.0(20–71)	40.90(9–71)
Temperature(˚C)	38.4(36.5–40.4)	37.9(36.5–40.7)	38.3(36.5–40.4)	38.15(36.5–40.7)
Hemoglobin(g/dl)	9.6(6.0–12.70)	11.1(5.34–14.7)	13.45(8.8–17.4)	12.4 (5.34–17.4)
GM Parasite(range)	14,801.04 (1200–57971)	10,972.71 (1000–142857)	11,301.58 (1200–150000)	11509.63 (1000–150000)
Gametocytemia (%)	0.00	3(11.5)	2(3.6)	5(5.5)
Previous malaria	4(44.4%)	13(50)	37(66.1)	54(59.3)
Bed net users	6(66.7%)	19(73.1)	22(39.3)	47(51.6)

**Abbreviations: GM**: geometric mean; **KG**: kilogram; **°C**: degree centigrade; **g/dl:** gram/deciliter

Average body temperature at recruitment was 38.15°C(36.5–40.7); 38.4°C(36.5–40.4) in U5, 37.9°C(36.5–40.7) in 5–15 years and 38.3°C (36.5–40.4) in above 15 yrs. At inclusion, higher average parasite load was observed in U5. Geometric mean of parasitemia at day-0 was 11,509.63 (1000–150,000) parasite/μl among all study participants: 14,801.04/μl (1200–57,971) in U5; 10,972.71/μl (1000–142857) in 5–15 yrs; and 11,301.58 (1200–150,000) in above 15 years. Gametocyte carriage was observed only among the older age groups; no gametocyte detected in U5. Hemoglobin level was significantly increased with age as placed in Table 1.

Previous malaria exposure was seen by about 60% of the study participants. The proportion increased with age where adults had higher frequency of malaria attack, compared to lower age. The mass distribution of ITNs, IRS and adoption of ACTs contributed much for the substantial decline in malaria related deaths in Ethiopia [24,25]. However, the current study findings indicated that still there were low awareness to the use of ITN; about half of the study participants were not using ITN (Table 1).

### Study Outcome: Primary and Secondary Endpoints

#### Cure Rate of Coartem®

**Fig 2:**
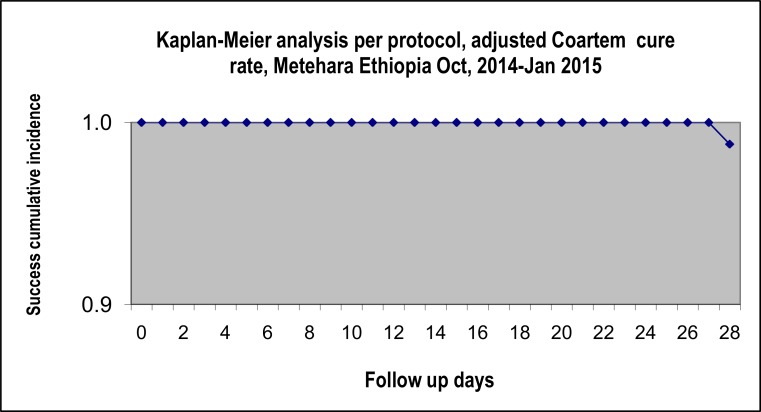
Survival analysis of 28-day cure rate of Coartem®, Metehara, Oct 2014-Jan 2015.

Of the 91 patients enrolled, 83 completed the follow-up at day-28 with ACPR. Two treatment failures, 1 LPF at day 23 and 1 LCF at day 28, were observed, giving PCR-uncorrected failure rate of 2.4% (2/83) (95% CI: 0.0–8.5%). By PCR correction, only the LCF was confirmed to be a recrudescence case with a failure rate of 1.2%. There was seen no early treatment failure (ETF). For per protocol (PP) analysis, PCR-uncorrected cure rate of Coartem® among the study participants was 97.6% (95%CI: 93.6–99.5) and the PCR-corrected cure rate was 98.8% (95%CI: 93.5–100%) (Fig 2 & Table 2). For intention to treat (ITT) analysis, the PCR-uncorrected cure rate was 91.2% (95%CI: 85.6–93.1%) and the PCR-corrected cure rate was 92.2% (86.2–96.1) (Table 2). ACPR at day-28 or adjusted cure rate was 100% each for U5 children and adults; whereas in 5–15 yrs, it was 92.3% (24/26) as shown in Table 2.

**Table 2:** The 28-day cure rate of Coartem®, Metehara, Ethiopia, Oct 2014 to Jan 2015.

	U5 yrs	5–15 yrs	>15 yrs	Total
Classification	No (%)	No (%)	No (%)	No (%)
Censored by LFU & WTH	0	0	6	6
ETF	0	0	0	0
LCF	0	1(4.17)	0	1(1.2)*
LPF	0	1(4.17)	0	1(1.2)#
ACPR at day 28	9(100)	24(91.7)	50(100)	83
PP PCR-uncorrected cure rate (95% CI)	NA	NA	NA	83/85; 97.6 (93.6–99.5)
PP PCR-corrected cure rate (95% CI)	NA	NA	NA	83/84; 98.8 (93.5–100)
ITT PCR-uncorrected cure rate (95% CI)	NA	NA	NA	83/91; 91.2 (84.5–96.3)
ITT PCR-corrected cure rate (95% CI)	NA	NA	NA	83/90; 92.2 (86.2–96.1)

**Abbreviations: U5:** under 5 years; **yrs:** years; **n:** sample per age; **No**: Number; **%**: percent; **ETF**: Early treatment failure; **LCF**: Late clinical failure; **LPF**: Late parasitological failure; **ACPR**: Adequate clinical and parasitological response; **CI**: confidence interval; **PP**: per protocol analysis; **ITT:** intention to treat analysis; **LFU**: loss to follow up; NA: Not applicable; **WTH**: withdrawal

* stands for recrudescence

# stands for new infection.

**Note**: 1) Nominator is the ACPR at day 28; 2) For PP, denominator (D) = Failure+ACPR_28_ for uncorrected & D = Recrudescence+ACPR_28 for_ corrected; 3) For ITT, D = enrolled for uncorrected, & D excludes new infections for corrected

#### Parasite clearance rate

At recruitment, 54.9% (50/91) of the study participants were severely parasitemic (>10,000 parasites/μl), while the rest 45.1% (41/91) were found moderately parasitemic (1000–9999 parasites/μl). High proportion of severe parasite density was observed in U5 children referred to older age groups. More detail on parasite density among the age groups is available on the Table 3.

**Table 3:** Degree of parasitemia among study participants, Metehara, Oct 2014-Jan 2015.

	U5	5–15 yrs	>15 yrs	Total
Parasitemia at recruitment	n (%)	n (%)	n (%)	n (%)
Moderate(1000–9,999/μl)	3(33.3%)	12(46.2%)	26(46.4%)	41(45.1%)
Severe(>10,000/μl)	6(66.7%)	14(53.8%)	30(53.6%)	50(54.9%)
Total	9(100.0%)	26(100.0%)	56(100.0%)	91(100.0%)

**Abbreviations: U5:** under 5 years; **yrs:** years; **n:** sample per age; **No**: Number; **%**: percent

**Fig 3:**
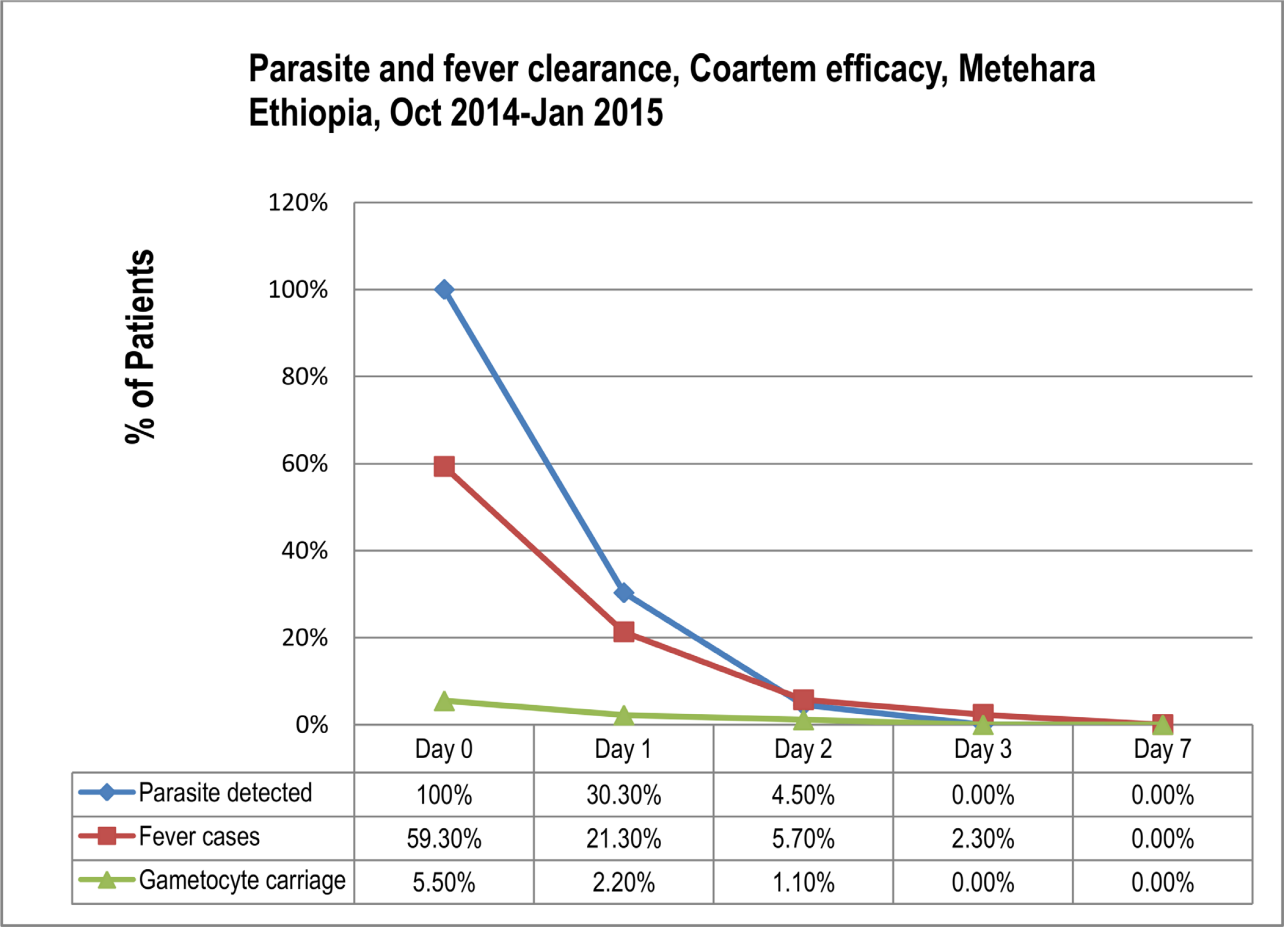
Parasite and fever clearance in Coartem efficacy study, Metehara, Oct 2014-Jan 2015.

In the current study, parasitemia cleared rapidly after medication of the patients: i.e. 69.7% clearance on day-1, 95.5% on day-2 and all parasitemia cleared on day 3 (Fig 3 & Table 4).

**Table 4:** Parasite and fever clearance rate in study participants, Metehara, Oct-Jan 2014/5.

Follow-up days		Day 0	Day 1	Day 2	Day 3
Patients attended	Age	n = 91	n = 89	n = 88	n = 87
Parasitemia Detected (%)	U5 yr	9(100)	5(55.6)	2(22.2)	-
	5–15 yr	26(100)	6(24.0)	1(4.0)	-
	>15 yr	56(100)	16(29.1)	1(1.9)	-
	Total	91(100)	27(30.3)	4(4.5)	-
Gametocyte Carriage (%)	5–15 yr	3(11.5)	2(8.0)	1(4.0)	-
	>15 yr	2(3.6)	-	-	-
	Total	5(5.5)	2(2.2)	1(1.1)	-
Fever Cases (≥37.5°C) (%)	U5 yr	6(66.7)	3(33.3)	1(11.1)	1(11.1)
	5–15 yr	13(50.0)	4(16.0)	1(4.0)	-
	>15 yr	35(62.5)	12(21.8)	3(5.6)	1(1.8)
	Total	54(59.3)	19(21.3)	5(5.7)	2(2.3)

**Abbreviations**: **°C**: degree centigrade; **n**: patients’ number; **yr**: year

#### Fever clearance rate

Febrile individuals, with ≥37.5°c axillary temperature, accounted for 59.3% (54/91) at the day of recruitment and decreased to 21.3% (19/89) on day 1, 5.7% (5/88) on day 2 and 2.3% (2/87) on days 3. Accordingly, fever clearance was 94.3% (83/88) on day 2, 97.7% (82/87) on day 3 and no febrile case was detected onwards (Fig 3 & Table 4). Fever case among severely parasitemic patients was 57.4% (31/54) and among the moderately parasitemic individuals was 42.6% (23/54). However, the difference between degree of parasitemia and fever case is not significant (P-value = 0.567) (Table 5).

**Table 5:** An association between parasitemia and fever, Metehara, Oct-Jan 2014/5.

	Fever Day 0			P-value
Parasitemia at enrolment	No fever	Fever case	Total	
Moderate	48.6%(18/38)	42.6%(23/54)	45.1%(41/91)	
Severe	51.4%(19/37)	57.4%(31/54)	54.9%(50/91)	
Total	100.0%(37/37)	100.0%(54/91)	100.0%(91/91)	0.567

#### Gametocytemia Clearance

Only 5.5% (5/91) gametocyte cases, among all study participants, were detected at enrolment: 11.5% (3/26) detected in 5–15 yrs, 3.6% (2/56) detected in >15 years and none [0.0% (0/9)] was detected in U5 age group. Of the day-0 detected gametocyte cases, three cases on day-1 and/or four cases on day-2 were cleared giving gametocytemia clearance rate of 60%(3/5) and 80%(4/5) on these days, respectively. The proportion of gametocyte carriage per total study participants was declined from 5.5% (5/91) on day 0 to 2.25% (2/89) on day 1, 1.14% (1/88) on day 2 and completely disappeared on day 3 (Fig 3 & Table 4). No new gametocyte case was detected after initiation of the treatment.

### Assessment of Hemoglobin Level and Anemia Classification

The proportion of anemia was decreased from 37.4% (34/91) on day 0 to 31.4% (27/86) on day 14 and/or 23.2% (19/82) on day 28. The mean hemoglobin of the study participants was significantly increased (P<0.000) from 12.39 g/dl (5.30–17.40g/dl) on day 0 to 12.85g/dl (7.00–17.10`g/dl) on day 14 and 13.45 g/dl (7.70–17.50g/dl) on day 28 (Table 6).

**Table 6:** Mean difference in hemoglobin among participants, Metehara, Oct-Jan 2014/5.

Hemoglobin	t	df	Sig(2-tailed)	Mean difference	95% CI (Lower—upper)
Hb day 0	45.064	90	.000	12.40044	11.8538–12.9471
Hb day 14	51.861	85	.000	12.84884	12.3562–13.3414
Hb day 28	58.455	81	.000	13.45610	12.9981–13.9141

Where **Hb**: hemoglobin; **t**: t-test; **df:** degree of freedom; **Sig:** significance

### Adverse Drug Reactions

No serious adverse event was observed throughout the 28 days follow up. Most of the adverse reactions to Coartem®, observed in this study, were minor as noticed by the manufacturer. Most reported cough, headache, weakness, joint pain, abdominal pain, and anorexia; and some reported ulceration of mouth area. There were observed 3.29% oral ulcerations and 8.79% cough cases on days 1 to day 3. These minor symptoms were soon disappeared with parasite clearance (data not shown)

## Discussion

In response to the resistance of malaria parasites to older antimalarials, WHO has brought the ACTs for global use as the first line drug for uncomplicated falciparum malaria in 2001. Accordingly, the use of AL as the first line drug for uncomplicated falciparum malaria in Ethiopia has been started in 2004 [1]. Since then, no more significant ACT resistant *P*. *falciparum* cases were reported in the country [10,18,26]. The current study similarly showed well tolerance and high efficacy of AL against uncomplicated malaria in Eastern Ethiopia. In reverse to the Thailand-Cambodian fake antimalarial free marketing trend and the continued use of artemisinin mono-therapy, whichhave brought AL resistance in their area [27,28], AL distribution in Ethiopia occurs only in the government sectors and this has potentially minimized the distribution of counterfeit drugs in the country [1].

The present study showed the 28 day cure rate of a standard six-dose of Coartem® as close to 100% in treating uncomplicated *P*. *falciparum* malaria, with rapid clearance of fever and parasitaemia within the first three days. For PP analysis, PCR-uncorrected & the PCR-corrected cure rate of Coartem® among the study participants were 97.6% (95%CI: 93.6–99.5) and 98.8% (95%CI: 93.5–100%), respectively. Similar high efficacy findings were already reported elsewhere in Ethiopia [8,10,19,29], and in sub-Saharan Africa [30,31]. Pooled-analysis in Coartem® cure rate from above 32 publications reported the average efficacy of 97% [14], which is almost similar to the present study finding.

According to the Kaplan Meier PP survival analysis in the current study, the PCR-uncorrected/corrected failure rate was 1.2–2.4%. Change in the national antimalarial treatment policy is done only if treatment failure rate is above 10% as recommended by WHO [16]. Therefore, this study finding advocates the continuation of AL as a first-line treatment of uncomplicated *P*. *falciparum* in the study area, with no change in the national treatment policy.

The adjusted cure rate at day-28 was 100% each for U5 children and adults; whereas in 5 to 15 years, it was 92.3%. Treatment failure in the present study was observed only in young ages that have yet not developed sufficient acquired immunity to malaria. The difference in cure rate among age groups may be due to the development of B-cell memory to malaria by old ages from frequent past infections that augments anti-malarial therapeutics to attain better cure rate than the non-immune young children [32,33]. The difference from U5 children may be due to the confounding attributed to the smaller sample size of under fives in the current study.

Parasitemia cleared rapidly on day-3 in this study, which is similar to the earlier study reports from Ethiopia [8,10,19,29] and India [34]. ACT is known by its rapid parasite clearance and reducing gametocyte carriage to end up parasite transmission [11–13]. AL is known to decrease both asexual and sexual stages of parasites 6 to 8 times faster than the previous anti-malarial drugs [35,36]. This shows the potent anti-malarial property of Coartem® targeting the blood stage parasites. No gametocyte case was detected on day 3 and onwards. Similar studies from different parts of Africa including Ethiopia [8,19,37] had reported the rapid clearance of gametocytes with Coartem® treatment.

Fever cleared rapidly following Coartem® treatment in the present study, and this is comparable to findings described elsewhere [8,18,19]. There was observed a minimal fever cases delayed one day longer after the clearance of parasitemia, which may be due to the metabolic end products from malaria parasite and the blood circulating malaria toxins that have prolonged half-life [38].

The mean hemoglobin of the study participants was significantly increased (P<0.000) from 12.39 g/dl on day 0 to 13.45 g/dl on day 28. This enhancement next to medication with Coartem® is analogous to other study reports [10,19,29]. Besides, hemoglobin level significantly increased with age from enrolment to completion of the study (P-value<0.05). This difference in proportion of anemia among age groups may be due to insufficient nutrition and limited immune defense in lower age groups than the higher ones as indicated somewhere [39].

There were observed no serious adverse events; most of the observed reactions were already noticed as common adverse reactions by the manufacturer and registered under the food and drug administration authority. Majority reported cough, headache, weakness, joint pain, abdominal pain, and anorexia; and some reported ulceration of mouth area. Those minor symptoms observed were soon disappeared with parasite clearance. The absence of any serious adverse event following Coartem® treatment in the current study matches the findings of other studies [19,30,31]. This still implies the safe continuation of Coartem® use as first–line treatment in the study district.

The relative bioavailability of artemether and lumefantrine increases by 2–3 times and 16 times, respectively, when administered after a high-fat meal [40]. However, in the current study, AL was administered without giving fatty food; only peanuts to adults and biscuits to children were provided preceding drug intake in the outpatient. This is one of the possible limitations in this study.

## Conclusion

The current result showed the high efficacy of Coartem® that suggests the continuation of the drug as first-line for the treatment of uncomplicated *P*. *falciparum* malaria in the study area. Even after a number of years of its widespread access in the country, the AL still works highly efficacious medication for uncomplicated *P*. *falciparum* infection in the study area. No similar study has been conducted in the study area prior to the current study; therefore, regular monitoring of the efficacy of the drug in the study area is recommended to handle the raising drug resistance issues early. This study recommends further study on drug toxicity, particularly on repeated cough and oral ulceration.

## Abbreviations

ACPR: Adequate clinical and parasitological response
ACT: Artemisinin-based combination therapy
AL: Artemether-lumefantrine
ETF: Early treatment failure
LCF: Late clinical failure
LPF: Late parasitological failure
WHO: World Health Organization
PCR: polymerase chain reaction
EPHI: Ethiopian public health institute
Hb: hemoglobin
ITNs: insecticide-treated bed nets
IRS: indoor residual spraying
WBCs: White blood cells
FMOH: Federal ministry of health
OPD: outpatient department
FCT: Fever Clearance Time
PCT: Parasite Clearance Time
ITT: Intention to treat
PP: Per protocol
U5: Under five children
